# Cost of postoperative sepsis in Vietnam

**DOI:** 10.1038/s41598-022-08881-y

**Published:** 2022-03-22

**Authors:** My Hanh Bui, Quynh Long Khuong, Phuong Anh Le, The Anh Nguyen, Quoc Hung Doan, Tuan Duc Duong, Hoang Ha Pham, Thanh Viet Pham, Tien Hung Tran, Hong Ha Nguyen, Binh Giang Tran, Duc Hung Duong, Xuan Co Dao, Gia Du Hoang, Xuan Thanh Dao, Truong Son Nguyen, Quang Cuong Le

**Affiliations:** 1 Hanoi Medical University, 1 Ton That Tung, Dong Da, Hanoi, 100000 Vietnam; 2 Hanoi Medical University Hospital, 1 Ton That Tung, Dong Da, Hanoi, 100000 Vietnam; 3 Hanoi University of Public Health, 1A Duc Thang, North Tu Liem, Hanoi, 100000 Vietnam; 4 Brandeis University, 415 South Street, Waltham, MA 02453 USA; 5 Friendship Hospital, 1 Tran Khanh Du, Hai Ba Trung, Hanoi, 100000 Vietnam; 6 Viet Duc Hospital, 33 Trang Thi, Hoan Kiem, Hanoi, 100000 Vietnam; 7 Vietnam Social Insurance Agency, 7 Trang Thi, Hoan Kiem, Hanoi, 100000 Vietnam; 8 Cho Ray Hospital, 201B Nguyen Chi Thanh, District 5, Ho Chi Minh City, 70000 Vietnam; 9 Bach Mai Hospital, 33 Giai Phong, Dong Da, Hanoi, 100000 Vietnam

**Keywords:** Outcomes research, Health care economics

## Abstract

Despite improvements in medical care, the burden of sepsis remains high. In this study, we evaluated the incremental cost associated with postoperative sepsis and the impact of postoperative sepsis on clinical outcomes among surgical patients in Vietnam. We used the national database that contained 1,241,893 surgical patients undergoing seven types of surgery. We controlled the balance between the groups of patients using propensity score matching method. Generalized gamma regression and logistic regression were utilized to estimate incremental cost, readmission, and reexamination associated with postoperative sepsis. The average incremental cost associated with postoperative sepsis was 724.1 USD (95% CI 553.7–891.7) for the 30 days after surgery, which is equivalent to 28.2% of the per capita GDP in Vietnam in 2018. The highest incremental cost was found in patients undergoing cardiothoracic surgery, at 2,897 USD (95% CI 530.7–5263.2). Postoperative sepsis increased patient odds of readmission (OR = 6.40; 95% CI 6.06–6.76), reexamination (OR = 1.67; 95% CI 1.58–1.76), and also associated with 4.9 days longer of hospital length of stay among surgical patients. Creating appropriate prevention strategies for postoperative sepsis is extremely important, not only to improve the quality of health care but also to save health financial resources each year.

## Introduction

Sepsis is a life-threatening organ dysfunction caused by a dysregulated host response to an infection^1^. Despite improvements in medical care, the burden of sepsis remains high. A recent global study reported 48.9 million sepsis cases and 11 million sepsis-related deaths in 2017, accounting for approximately 20% of all annual deaths globally^2^. The mortality rate of sepsis is estimated to be 26.7% in hospitals and 42% in intensive care units (ICUs). Among adult sepsis survivors, more than 30% died within 1 year, and one in six experienced significant long-term morbidities, such as functional limitations, cognitive impairment, and mental health disorders^3^. Furthermore, sepsis is a costly condition, and it can increase treatment cost due to multiple organ dysfunction. A recent systematic review showed that the mean total hospital cost per patient treated for sepsis varied between 13,292 USD^4^ and 75,015 USD^5^ in high-income countries (HICs). It is estimated that sepsis contributes to an annual economic burden of more than 20 billion USD in the US^6^.

Postoperative complications account for approximately 30% of inpatient cases of severe sepsis^7^; however, evidence of the incremental cost associated with sepsis complications after surgery is scarce. Only a few studies exist, and these were conducted in HICs. A study in the US indicated that the cost of sepsis complications is 26,972 USD per case, which contributes to 2.28 times greater of treatment cost for patients with sepsis relative to patients without sepsis^8^. To our best knowledge, there have still been no studies conducted in low- and middle-income countries (LMICs), such as Vietnam, to investigate the incremental cost of postoperative sepsis. The absence of such information may result in a lack of a strategy to alleviate the economic burden of surgical complications in general, and sepsis complications specifically. This has now become an urgent issue, since much of the burden of sepsis, in terms of both incidence and mortality, is in LMICs^9^.

In this study, we use the Vietnam Social Insurance agency (VSI) national database of more than 1.2 million surgical patients to estimate the incremental cost of sepsis as a complication after major surgery in Vietnam. We also evaluate the impact of sepsis on other patient treatment outcomes, including readmission, reexamination, and hospital length of stay (LOS).

## Methods

### Data source and study participants

Vietnam is a developing country located in Southeast Asia, with a population of 96.5 million people^10^. In 2018, per capita GDP was 2,566 USD, with total healthcare expenditure of approximately 17.2 billion USD and health expenditure per capita of 152 USD (5.9% GDP)^11^.

In this study, we used hospital discharge records from the national electronic payment portal database developed by the VSI. The VSI is the largest database of hospitalization records, including all payer payments in Vietnam, covering 87% of Vietnamese population across 63 provinces. The VSI manages the information on patient diagnoses and discharge diagnosis codes using the International Classification of Diseases, 10th revision (ICD-10). For this study, we extracted all patient records from 1 January 2017, to 30 September 2018. We included surgical patients aged ≥ 18 who had undergone one of seven types of surgery: spinal-neurological; cardiothoracic; vascular; gastrointestinal; urological; orthopedic; and plastic surgery. A total of 1,241,893 surgical patients were included in the final study sample.

### Sepsis definition

In the VSI data, sepsis includes sepsis and septic shock within 30 days of surgery. Sepsis is diagnosed based on evidence of infection detected by blood cultures plus evidence of systemic inflammatory response syndrome (SIRS), manifested by two or more of the following conditions: (1) temperature > 38 °C or < 36 °C; (2) heart rate > 90/min; (3) respiratory rate > 20/min or PaCO2 < 32 mm Hg (4.3 kPa); white blood cell count > 12,000/mm^3^ or < 4,000/mm^3^ or > 10% immature bands^1^. Septic shock is identified if a patient meets the criteria for sepsis, and also has circulatory and metabolic abnormalities, including persistent hypotension despite adequate volume resuscitation combined with perfusion abnormalities, such as lactic acidosis or oliguria^1^.

### Outcomes

#### Direct cost

The primary outcome is the cost of sepsis complications associated with the index admission and all subsequent events within 30 days of discharge, including all hospital and readmission payments, physician payments, outpatient payments, and prescription drug payments. The costs are the direct medical costs collected based on a bottom-up approach, which is the total amount of payment for the entire episode of treatment. All costs are presented as USD, with 1 USD = 23,255 Vietnamese dong as of September 2018.

#### Readmission, reexamination, and LOS

The secondary outcomes of this study include (1) readmission, (2) reexamination, and (3) LOS. Readmission refers to any overnight stay at an inpatient hospital within 30 days of the index discharge. Reexamination refers to any visit to an inpatient hospital, outpatient hospital, emergency department, or other healthcare center to reexamine postoperative problems. LOS is the total number of days of treatment as an inpatient. We assessed both the LOS of the index admission (from date of surgery to discharge) and total LOS at 30 days, which is calculated as the LOS of the index admission plus LOS of readmission within 30 days of surgery.

### Covariates

The covariates include patient social-demographic characteristics, including age, gender, place of residence, and emergency hospitalization. We also evaluated the 21 preoperative comorbidities based on the list of chronic comorbidities introduced by Elixhauser et al.^12^.

### Statistical analysis

We used Chi-squared tests or the Fisher’s exact tests and t-tests to examine the balance in patient characteristics between the two groups; those patients having sepsis, and those having no sepsis.

#### The incremental cost of sepsis

We estimated the cost from the healthcare perspective, which includes both payments by health insurance and out-of-pocket payments by patients. We used generalized linear models to estimate the cost of treatment and incremental cost due to sepsis complications. The link function was specified as a log. Since the cost data were inherently positively skewed, we applied gamma distribution^13,14^.

Before estimating the incremental cost of sepsis, we excluded patients with multiple complications; i.e., we compared patients with no sepsis with those with sepsis complications only. This approach helps to avoid a compounding effect and achieves a more precise estimate of the burden attributable to sepsis complications. To obtain the balance in patient characteristics between the two groups, we applied a propensity score matching method. We conducted 1:1 ratio matching using nearest neighbor algorithms with a caliper of 0.1 standard deviations of the logit scale of the propensity score, to match one patient with sepsis with another patient with no sepsis who had the closest propensity scores. The propensity scores were calculated based on patient socio-demographics, emergency hospitalization status, and the list of 21 preoperative comorbidities.

To isolate the incremental cost of sepsis in the specific type of surgery, we applied a doubly robust estimation. This approach is described elsewhere^15,16^. The 95% confidence intervals (95% CI) were estimated based on a bootstrapping method, with 1000 replications. The same procedure was applied for estimating the incremental LOS, except that a Poisson distribution was used, reflecting the nature of the count data.

#### Impact of sepsis on readmission and reexamination

Logistic regressions were carried out to evaluate the effect of sepsis on readmission and reexamination of patients within 30 days of surgery. The models were adjusted for patient socio-demographic and hospital characteristics, emergency hospitalization status, and preoperative comorbidities.

A significance level of 0.05 was used for all statistical tests. All analyses were conducted using Stata v16 (StataCorp, College Station, TX, USA).

### Ethical consideration

All procedures performed in this study were in accordance with the ethical standards of the Ethical Review Board of Hanoi Medical University (IRB approval No. 67/HDDDDHYHN; Dated: March 24, 2017). The data were obtained under permission from the Vietnam Social Insurance Agency. All patient information is anonymous.

## Results

### Study sample

**Figure 1:**
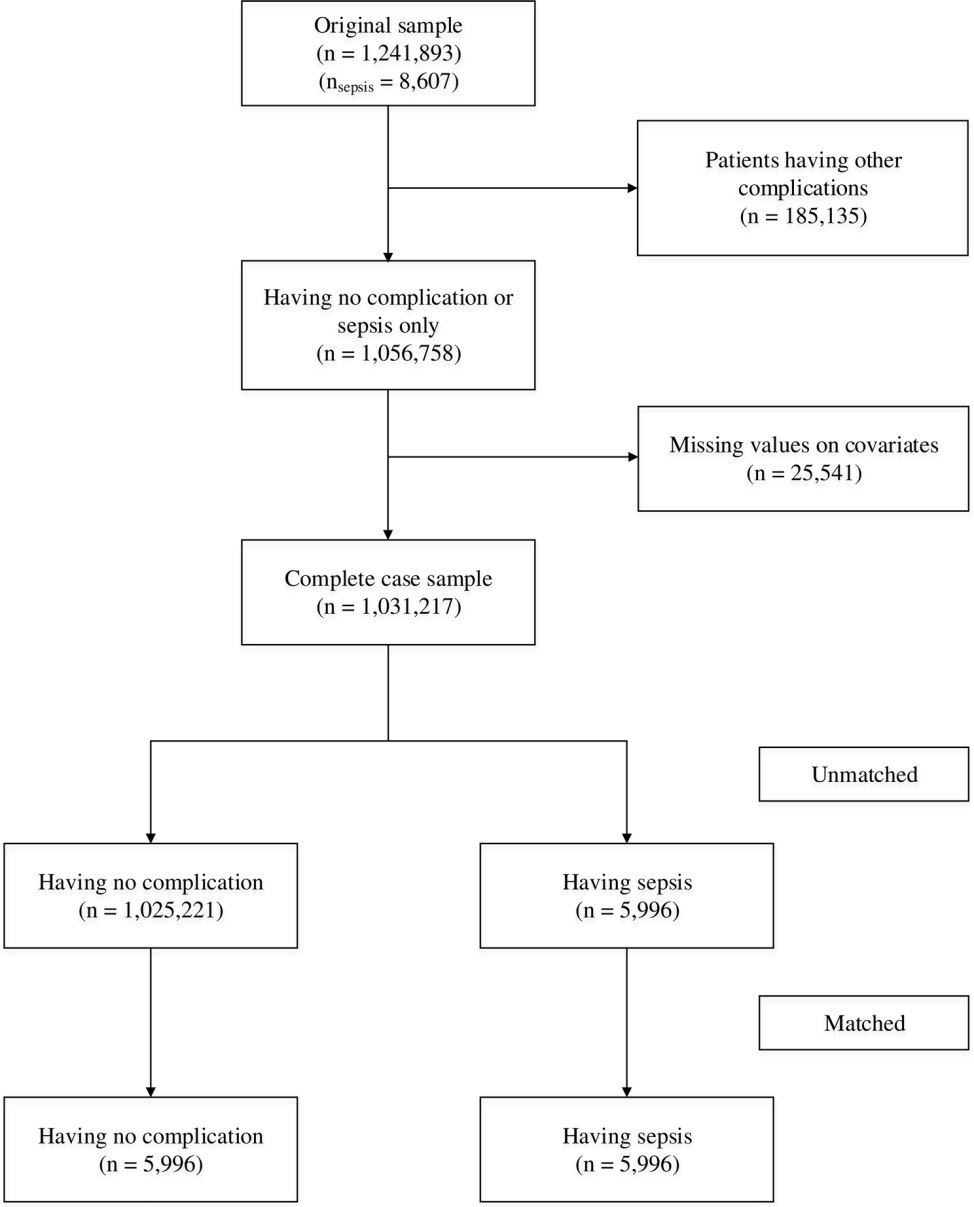
Flow diagram of the study sample size.

Figure 1 provides information on the sample size of this study. The original sample size was 1,241,893 surgical patients, of whom 8607 patients developed sepsis after surgery (0.69%). After excluding patients with multiple complications and those with missing covariates (2.42%), the complete case sample was 1,031,217 (n sepsis = 5,996) patients.

### Matching

Table 1 summarizes the preoperative characteristics of surgical patients with sepsis before and after matching. We matched 5996 patients with sepsis with 5996 patients with no sepsis. There were no significant differences in patient characteristics between the two groups except for peripheral vascular disease.

**Table 1:** Baseline characteristics of surgical patients by sepsis before and after matching.

Factors	No sepsis		Sepsis n (%)	P-value^a^	
	Raw sample n (%)	Matched sample n (%)		Raw sample	Matched sample
N	1,025,221	5,996	5,996		
Age, mean (SD)	46.9 (17.3)	52.5 (18.0)	52.3 (17.9)	< 0.001^b^	0.65 ^b^
**Gender**					
Male	613,861 (59.9)	3747 (62.5)	3733 (62.3)	< 0.001	0.79
Female	411,360 (40.1)	2249 (37.5)	2263 (37.7)		
**Region**					
Northern Midlands and Mountainous	134,976 (13.2)	1031 (17.2)	1010 (16.8)	< 0.001	0.98
Red River Delta	229,401 (22.4)	882 (14.7)	879 (14.7)		
North Central and South Central Coast	259,894 (25.4)	1825 (30.4)	1808 (30.2)		
Central Highland	60,444 (5.9)	240 (4.0)	244 (4.1)		
Southeast	182,402 (17.8)	646 (10.8)	662 (11.0)		
Mekong River Delta	158,104 (15.4)	1372 (22.9)	1393 (23.2)		
Emergency hospitalization	196,872 (19.2)	1253 (20.9)	1310 (21.8)		0.20
**Preoperative concomitant diseases**					
Heart failure	7055 (0.7)	95 (1.6)	96 (1.6)	< 0.001	0.94
Valvular heart disease	3940 (0.4)	33 (0.6)	29 (0.5)	0.22	0.61
Peripheral vascular disease	2109 (0.2)	30 (0.5)	49 (0.8)	< 0.001	0.032
Arrhythmia	5999 (0.6)	52 (0.9)	63 (1.1)	< 0.001	0.30
Hypertension	91,907 (9.0)	947 (15.8)	959 (16.0)	< 0.001	0.76
Cerebrovascular disease	25,476 (2.5)	235 (3.9)	246 (4.1)	< 0.001	0.61
Paralysis	986 (0.1)	9 (0.2)	8 (0.1)	0.35	0.81
Chronic lung disease	15,907 (1.6)	145 (2.4)	148 (2.5)	< 0.001	0.86
Diabetes	36,439 (3.6)	655 (10.9)	630 (10.5)	< 0.001	0.46
Complicated diabetes	583 (0.1)	34 (0.6)	41 (0.7)	< 0.001	0.42
Gastritis	104,258 (10.2)	814 (13.6)	825 (13.8)	< 0.001	0.77
Hypothyroidism	1231 (0.1)	8 (0.1)	9 (0.2)	0.50	0.81
Chronic renal failure	6069 (0.6)	124 (2.1)	120 (2.0)	< 0.001	0.80
Liver diseases	24,108 (2.4)	293 (4.9)	290 (4.8)	< 0.001	0.90
Metastatic cancer	4797 (0.5)	28 (0.5)	34 (0.6)	0.26	0.44
Cancer	44,609 (4.4)	213 (3.6)	220 (3.7)	< 0.001	0.73
Joint disease	11,616 (1.1)	132 (2.2)	132 (2.2)	< 0.001	1.00
Weight loss	8453 (0.8)	64 (1.1)	88 (1.5)	< 0.001	0.050
Fluid and Electrolyte Disorders	1476 (0.1)	29 (0.5)	34 (0.6)	< 0.001	0.53
Anemia	3483 (0.3)	32 (0.5)	46 (0.8)	< 0.001	0.11
Clotting disorder	556 (0.1)	6 (0.1)	7 (0.1)	0.049^c^	0.78
Depression/Addiction	585 (0.1)	0 (0.0)	0 (0.0)	0.064	–

^a^Chi-squared test unless otherwise stated.

^b^t test.

^c^Fisher's exact test.

### Costs of treatment by types of surgery and their predictors

The costs of each type of surgery and their predictors are shown in Supplemental Table 1 and Supplemental Table 2. The median unadjusted cost of the index admission was 337.2 USD (IQR: 212.1–674.9), and the total 30-day cost was 722.3 USD (IQR: 448.6–1454.5). The highest cost was found in patients undergoing vascular surgery, and the lowest cost was found in patients undergoing plastic surgery. After multivariable adjustment, several factors were found to be associated with higher treatment costs. Specifically, patients of older age, male patients, and patients with preexisting medical comorbidities are associated with higher treatment costs; both the cost of the index admission and the total 30-day cost.

### Incremental cost due to sepsis complications

The matched sample was used to estimate the incremental cost of sepsis complications (Table 2). Sepsis complications significantly increase both the cost of the index admission and the total 30-day cost. The incremental cost associated with sepsis complications was 279.9 USD (95% CI 214.1–352.9) for the index admission and 724.1 USD (95% CI 553.7–891.7) for the 30 days after surgery; this sum is equivalent to 28.2% of per capita GDP in Vietnam in 2018. The service costs of readmission contribute most to the incremental cost of sepsis complications, while reexamination was not a significant contributor to the incremental cost.

**Table 2:** Costs, length of hospital stay, and incremental estimates due to sepsis complication after surgery.

	Mean cost (USD)		Cost of sepsis, USD (% GDP^a^)	95% Bootstrap CI	P-value
	No sepsis	Sepsis			
N	5,996	5,996			
**Treatment cost**					
Cost of the index admission	878.5	1158.4	279.9 (10.9)	214.1–352.9	< 0.001
**30-day costs**					
Service cost for 30-day readmission	740.7	1008.2	267.4 (10.4)	209.6–332.0	< 0.001
Drug cost for 30-day readmission	159.8	328.1	168.3 (6.6)	136.0–206.1	< 0.001
Service cost for 30-day reexamination	47.3	52.9	5.5 (0.2)	− 2.3–17.1	0.195
Drug cost for 30-day reexamination	22.0	24.9	2.9 (0.1)	− 1.9–11.2	0.288
Total 30-day costs	1848.4	2572.6	724.1 (28.2)	553.7–891.7	< 0.001
**Difference in length of stay**					
Length of stay (days)					
Length of hospital stay	11.6	13.5	1.9	1.5–2.3	< 0.001
Total length of treatment within 30 days^†^	12.9	17.8	4.9	4.3–5.6	< 0.001

*95% CI* 95% Confidence interval; All estimates were calculated using a matched sample.

^**§**^Compared to GDP per capita in Vietnam in 2018 (2,566 USD).

^†^Includes length of hospital stay in the treatment and 30-day readmission periods.

Table 3 shows the incremental cost of sepsis complications by type of surgery. The highest incremental cost was found in patients undergoing cardiothoracic surgery, at 1,177.5 USD (95% CI 174.4–2180.7) for the index admission and 2,897 USD (95% CI 530.7–5263.2) for the 30-day total. In contrast, the lowest incremental cost of sepsis complication was associated with orthopedic surgery, at 235.9 USD (95% CI 25.2–132.6) and 78.9 USD (95% CI 116.7–355.1) for the index admission and 30-day total, respectively.

**Table 3:** Estimated incremental costs, by type of surgery and complications.

	Mean cost (USD)		Cost of Sepsis, USD (% GDP^a^)	95% Bootstrap CI	P-value
	No sepsis	Sepsis			
**Index admission cost, breakdown by types of surgery**					
Spinal-neurological (n matched = 179)	1734.9	2276.6	520.8 (20.3)	66.3–975.3	0.025
Cardiothoracic (n matched = 121)	1814.3	3097.1	1177.5 (45.9)	174.4–2180.7	0.021
Vascular (n matched = 75)	2978.3	3448.7	470 (18.3)	–	–
Gastrointestinal (n matched = 1625)	749.7	1364.1	611.3 (23.8)	504.6–717.9	< 0.001
Urological (n matched = 442)	668.7	1329.8	648.6 (25.3)	507.9–789.3	< 0.001
Orthopedic (n matched = 3251)	779.8	862.8	78.9 (3.1)	25.2–132.6	0.004
Plastic (n matched = 299)	476.7	951.7	532.8 (20.8)	322.5–743.2	< 0.001
**30-day costs, breakdown by types of surgery**					
Spinal-neurological (n matched = 179)	3660.6	5002.2	1299.4 (50.6)	234.2–2364.5	0.017
Cardiothoracic (n matched = 121)	4027.6	7158.1	2897 (112.9)	530.7–5263.2	0.016
Vascular (n matched = 75)	6121.2	6857.5	730 (28.4)	–	–
Gastrointestinal (n matched = 1625)	1609.2	3101.2	1485.2 (57.9)	1240.0–1730.4	< 0.001
Urological (n matched = 442)	1452.8	2927.5	1443.2 (56.2)	1131.4–1755.0	< 0.001
Orthopedic (n matched = 3251)	1645.0	1890.4	235.9 (9.2)	116.7–355.1	< 0.001
Plastic (n matched = 299)	1045.3	2153.5	1242.9 (48.4)	736.1–1749.8	< 0.001

*95% CI* 95% Confidence interval; All estimates were calculated using a matched sample.

^**§**^Compared to GDP per capita in Vietnam in 2018 (2,566 USD).

### Impact of sepsis complication on readmission, reexamination, and LOS of patients

Table 2 and Supplemental Table 3 show the impact of sepsis complications on secondary outcomes, including readmission, reexamination, and LOS of patients. Sepsis complications significantly increased the LOS of patients, at an average 1.9 days and 4.9 days for the index admission and the 30-day period after surgery, respectively (Table 2).

After controlling for patient socio-demographics, emergency hospitalization status, and preoperative comorbidities, patients with sepsis complications had 6.40 (95% CI 6.06–6.76) times higher odds of readmission as compared to patients without sepsis. Sepsis complications were also associated with an increase of 1.67 (95% CI 1.58–1.76) times the odds of reexamination of postoperative problems within 30 days of surgery (Supplemental Table 3).

## Discussion

This is the first study in Vietnam to estimate the cost of postoperative sepsis, using national data of 1,241,893 surgical patients who had undergone one of seven main types of surgery. We found that sepsis complications significantly increase the cost of treatment. The average incremental cost associated with sepsis complications was 724.1 USD for the 30 days after surgery; this sum is equivalent to 28.2% of per capita GDP in Vietnam in 2018. The highest incremental cost was found in patients undergoing cardiothoracic surgery, at 2897 USD. Surgical patients with sepsis also had higher odds of readmission, reexamination, and longer LOS.

In the literature, there is a lack of evidence on the economic burden of sepsis, although several studies have estimated the cost of sepsis treatment^4,17–21^. However, all of these studies were conducted in HICs. Furthermore, the cost estimates vary widely between countries and are hard to compare since health systems, patient inclusion criteria, cost structures, and cost estimation methods are very different. Information relating to incremental costs due to sepsis complications after surgery is even more scarce. A rare study to investigate the incremental cost of sepsis as a complication after surgery in the US indicated that the average unadjusted cost for patients with sepsis was 3.6 times higher than for patients with no sepsis. This number in risk-adjusted analyses was 2.28 times, which corresponds to 26,972 USD^8^. In our study, the incremental cost of sepsis as a postoperative complication is smaller than in the US study. This seems obvious, since healthcare systems and expenditure are very different between a LMIC and a HIC. Although the absolute cost relating to sepsis in our study is 724.1 USD, which is relatively small as compared to HICs, the sum is huge when compared to the per capita GDP of Vietnamese patients. In particular, the incremental cost of 28% per capita GDP is greater than the threshold of catastrophic health expenditure, which is defined as health expenditure that is greater than 25% of total household expenditure^22^. It is noticeable that the incremental cost of sepsis varies significantly between types of surgery. For example, the incremental cost in cardiothoracic surgery is 2897 USD, which is higher than per capita GDP in Vietnam; the incremental cost in orthopedic surgery is 235.9 USD. This difference can be explained because the condition of patients undergoing cardiothoracic surgery is usually more complex than those undergoing orthopedic surgery, and thus cardiothoracic patients generally have a higher risk of multiple organ dysfunction and septic shock^23,24^.

Besides the economic burden of sepsis complications, we also found that sepsis increased the odds of readmission, reexamination, and LOS of surgical patients. These findings are consistent with studies in other countries. Previous studies have indicated readmission rates related to sepsis ranging from 18–26% across clinical settings^25–29^, with most cases occurring within 30 days of discharge^25,26,30^. A study in the US indicated that the LOS of patients with positive blood culture results was longer than four days, as compared to those having negative results^31^. Furthermore, an increase in LOS and the risk of readmission and reexamination also result in increased healthcare costs. It is estimated that 30-day sepsis-related readmissions each cost around 25,000–30,000 USD and contribute to a cost of 1.4 billion USD annually in the US^25,30^. Together, the findings indicate that the cost burden of sepsis is substantial regardless of the context of the healthcare system, both in HICs and LMICs. Thus, creating appropriate prevention strategies for sepsis is extremely important, not only to improve healthcare quality but also to save considerable financial sums each year.

Although the burden of sepsis is substantial, approximately 20% of all-cause global deaths are due to sepsis and are largely preventable^9^. Preventing sepsis is an integral part of achieving UN Sustainable Development Goals, particularly targets 3.1, 3.2, 3.3, and 3.8^9^. The 70th World Health Assembly in May 2017 endorsed resolution 70.7 on *“Improving the prevention, diagnosis and clinical management of sepsis”*^32^, which urged the WHO to develop guidelines on sepsis prevention and management and to gather information on global epidemiology and the impact of the burden of sepsis^32^. However, global estimates of the burden of sepsis cases are hampered by the scarcity of data, in particular data from LMICs^9^. The Global Surgery 2030 targets include protection against impoverishing health expenditures^33^; however, many challenges exist to the accurate and comprehensive measurement of the global burden of disease due to the scarcity of data among LMICs^33^. Therefore, our study may provide important information for developing cost-effective prevention strategies for sepsis and contribute to the global evidence on the burden of sepsis, especially in the context of LMICs.

### Strengths and limitations

The strengths of this study include the nationally representative data with a large sample size. This study is one of the very few studies to investigate the incremental cost of sepsis as a complication after surgery. Using a strong analytical approach, we were able to obtain precise estimates of the incremental cost due to postoperative sepsis. However, the findings of this study should be interpreted in the context of potential limitations. First, we calculated only the direct medical cost, which underestimates the economic burden of postoperative sepsis. Second, to calculate the incremental cost of sepsis, we had to exclude patients with multiple complications; however, this may have the potential of underestimating the cost, since sepsis can cause other complications which increase treatment costs. Third, although social-demographic characteristics, emergency hospital, and comorbidities were used as covariates to control for the balance between groups, some factors can also affect the relationship between sepsis complication and hospital costs, such as hospital quality. Fourth, our study applied the sepsis definition which requires laboratory confirmation by blood culture rather than evidence of SIRS alone. Although the blood culture bacteriologically confirms the diagnosis, there are potential of some patients with sepsis having no results of blood culture. Finally, we cannot track those patients seeking self-treatment in drug counters and pharmacies.

## Conclusions

The incremental cost of postoperative sepsis is substantial. Sepsis also increases the LOS, and the odds of readmission and reexamination of surgical patients. Creating appropriate prevention strategies for postoperative sepsis is extremely important, not only to improve the quality of healthcare but also to save health financial resources each year.

## Supplementary Information

Supplementary Information.

## Data Availability

Some fields in data, data dictionary, statistical analysis plan, and code for the analysis are available for research purposes upon reasonable request through the corresponding author.
