# Supplementary material for: Cost of postoperative sepsis in Vietnam
Source: Sci Rep. 2022 Mar 22;12:4876. doi: 10.1038/s41598-022-08881-y (PMC8941147; doi:10.1038/s41598-022-08881-y)
Supplement: Supplementary file 1 — Supplementary Information. [file 41598_2022_8881_MOESM1_ESM.docx]

**Supplemental Table 1: Unadjusted cost of treatment by types of surgery.**

|  | **Index admission cost,**  **USD** | **Total 30-day cost,**  **USD** |
| --- | --- | --- |
| Overall, mean (SD) | 687.4 (1067.6) | 1446.1 (2233.8) |
| Overall, median (IQR) | 337.2 (212.1–674.9) | 722.3 (448.6–1454.5) |
| Spinal-neurological, mean (SD) | 1538.9 (1455.0) | 3238.0 (3056.3) |
| Spinal-neurological, median (IQR) | 1133.1 (501.0–2236.1) | 2437.0 (1073.9–4667.3) |
| Cardiothoracic, mean (SD) | 1858.7 (2235.1) | 3909.1 (4774.2) |
| Cardiothoracic, median (IQR) | 1086.9 (437.4– 2413.1) | 2348.6 (959.6–5005.9) |
| Vascular, mean (SD) | 2534.6 (3132.6) | 5034.1 (6325.0) |
| Vascular, median (IQR) | 1336.5 (325.9–3738.4) | 2782.1 (688.8–7193.9) |
| Gastrointestinal, mean (SD) | 624.8 (1034.2) | 1315.8 (2082.2) |
| Gastrointestinal, median (IQR) | 308.0 (224.4–549.0) | 669.5 (480.7–1184.4) |
| Urological, mean (SD) | 627.4 (830.1) | 1352.6 (1785.0) |
| Urological, median (IQR) | 466.9 (308.6–683.2) | 999.9 (653.3–1491.3) |
| Orthopedic, mean (SD) | 575.9 (796.7) | 1208.0 (1730.4) |
| Orthopedic, median (IQR) | 304.1 (189.7–601.4) | 643.5 (396.5–1284.2) |
| Plastic surgery, mean (SD) | 285.6 (657.3) | 601.6 (1384.9) |
| Plastic surgery, median (IQR) | 143.3 (60.8–281.9) | 294.6 (124.0–590.4) |

**Supplemental Table 2: Gamma regressions for predictors of index admission cost and total 30-day cost after surgery**

| **Factors** | **Cost of the index admission** | | | | | | **Total 30-day costs** | | | | | |
| --- | --- | --- | --- | --- | --- | --- | --- | --- | --- | --- | --- | --- |
|  | **Unadjusted** | | | **Adjusted^a^** | | | **Unadjusted** | | | **Adjusted^a^** | | |
|  | **Cost difference**  **(USD)** | **95%CI** | **P value** | **Cost difference**  **(USD)** | **95%CI** | **P value** | **Cost difference**  **(USD)** | **95%CI** | **P value** | **Cost difference**  **(USD)** | **95%CI** | **P value** |
| **Age** |  |  |  |  |  |  |  |  |  |  |  |  |
| 18-40 | Ref |  |  | Ref |  |  | Ref |  |  | Ref |  |  |
| 41-60 | 231.7 | 225.9–237.6 | <0.001 | 151.8 | 147.3–156.3 | <0.001 | 469.0 | 456.8–481.4 | <0.001 | 304.7 | 295.5–314.1 | <0.001 |
| 61-74 | 521.1 | 509.9–532.5 | <0.001 | 327.4 | 319.2–335.7 | <0.001 | 1061.2 | 1037.8–1084.9 | <0.001 | 656.8 | 640.2–673.6 | <0.001 |
| ≥75 | 587.4 | 571.8–603.3 | <0.001 | 449.6 | 437.0–461.8 | <0.001 | 1257.9 | 1224.2–1292.2 | <0.001 | 952.6 | 926.8–979.0 | <0.001 |
| **Gender** |  |  |  |  |  |  |  |  |  |  |  |  |
| Male | Ref |  |  | Ref |  |  | Ref |  |  | Ref |  |  |
| Female | -0.4 | -4.6–3.8 | 0.841 | -40.6 | -43.8–-37.3 | <0.001 | -10.0 | -18.6–-1.2 | 0.026 | -92.7 | -99.5–-85.8 | <0.001 |
| **Region** |  |  |  |  |  |  |  |  |  |  |  |  |
| Northern Midlands and Mountainous | Ref |  |  | Ref |  |  | Ref |  |  | Ref |  |  |
| Red River Delta | 213.9 | 203.8–24.2 | <0.001 | 118.2 | 110.8–125.9 | <0.001 | 432.3 | 411.3–453.8 | <0.001 | 231.9 | 216.5–247.6 | <0.001 |
| North Central and South Central Coast | 78.5 | 71.1–86.1 | <0.001 | 24.7 | 19.0–30.6 | <0.001 | 134.5 | 119.2–150.1 | <0.001 | 18.4 | 7.0–30.2 | 0.001 |
| Central Highland | -25.6 | -34.0–-17.0 | <0.001 | -17.0 | -24.6–-9.3 | <0.001 | -74.0 | -91.8–-56.0 | <0.001 | -63.4 | -78.3–-48 | <0.001 |
| Southeast | 3.5 | 75.5–91.8 | <0.001 | 45.4 | 38.9–52.1 | <0.001 | 156.2 | 139.5–173.3 | <0.001 | 65.1 | 52.0–78.6 | <0.001 |
| Mekong River Delta | 5.6 | -1.1–12.5 | 0.102 | -51.7 | -57.2–-46.2 | <0.001 | -5.5 | -19.8–8.8 | 0.444 | -136.8 | -146.8–-126.4 | <0.001 |
| **Emergency hospitalization** | -27.3 | -32.2–-22.3 | <0.001 | 31.2 | 26.2–36.1 | <0.001 | -18.6 | -29.3–-7.7 | 0.001 | 102.6 | 92.1–113.2 | <0.001 |
| **Preoperative concomitant diseases^b^** |  |  |  |  |  |  |  |  |  |  |  |  |
| Heart failure | 1645.2 | 1559.0–1734.9 | <0.001 | 588.8 | 546.6–633.3 | <0.001 | 3522.4 | 3339.9–3712.3 | <0.001 | 1264.7 | 1175.7–1357.1 | <0.001 |
| Valvular heart disease | 2350.6 | 2201.9–2507.1 | <0.001 | 1561.6 | 1460.9–1667 | <0.001 | 4944.2 | 4633.3–5271.4 | <0.001 | 3257.9 | 3051.0–3474.7 | <0.001 |
| Peripheral vascular disease | 1340.7 | 1209.0–1481.9 | <0.001 | 774.3 | 691.5–862.5 | <0.001 | 2886.4 | 2606.5–3186.3 | <0.001 | 1652.7 | 1479.2–1837 | <0.001 |
| Arrhythmia | 861.8 | 800.2–926.1 | <0.001 | 161.7 | 132.7–192.3 | <0.001 | 1866.4 | 1735.4–2003.1 | <0.001 | 357.9 | 296.8–421.4 | <0.001 |
| Hypertension | 464.3 | 451.4–477.4 | <0.001 | 96.2 | 88.8–104.3 | <0.001 | 992.4 | 965.1–1020.1 | <0.001 | 209.1 | 193.0–225.4 | <0.001 |
| Cerebrovascular disease | 355.1 | 334.4–376.3 | <0.001 | 94.3 | 81.2–107.5 | <0.001 | 791.2 | 747.0–836.6 | <0.001 | 222.8 | 194.8–251.4 | <0.001 |
| Paralysis | 1231.5 | 1051.7–1430.3 | <0.001 | 1054.8 | 911.3–1210.7 | <0.001 | 2683.54 | 2298.6–3108.9 | <0.001 | 2286.9 | 1984.8–2616.4 | <0.001 |
| Chronic lung disease | 475.1 | 446.1–505.0 | <0.001 | 144.8 | 126.8–163.0 | <0.001 | 1052.9 | 990.9–1116.8 | <0.001 | 334.8 | 297.3–373.3 | <0.001 |
| Diabetes | 570.6 | 549.1–592.5 | <0.001 | 195.4 | 182.2–208.7 | <0.001 | 1230.8 | 1185.4–1277.1 | <0.001 | 430.8 | 403.3–458.9 | <0.001 |
| Complicated diabetes | 778.2 | 608.2–971.1 | <0.001 | 140.1 | 56.2–233.6 | 0.001 | 1775.3 | 1403.3–2197.0 | <0.001 | 360.2 | 180.2–561.1 | <0.001 |
| Gastritis | 198.2 | 189.0–207.5 | <0.001 | 54.7 | 47.9–61.6 | <0.001 | 425.6 | 406.3–445.2 | <0.001 | 124.7 | 110.9–138.7 | <0.001 |
| Hypothyroidism | 209.8 | 135.0–291.7 | <0.001 | 41.2 | -11.6–98.6 | 0.131 | 408.7 | 255.0–577.1 | <0.001 | 73.2 | -35.2–190.5 | 0.191 |
| Chronic renal failure | 2077.6 | 1968.6–2191.2 | <0.001 | 1825.8 | 1736.9–1917.1 | <0.001 | 4239.7 | 4016.9–4472.0 | <0.001 | 3660.1 | 3483.2–3843.7 | <0.001 |
| Liver disease | 367.2 | 345.7–389.2 | <0.001 | 91 | 77.1–104.8 | <0.001 | 802.3 | 756.7–849.0 | <0.001 | 209.6 | 181.3–238.5 | <0.001 |
| Metastatic cancer | 1076.2 | 998.3–1158.0 | <0.001 | 616.9 | 566.7–670.1 | <0.001 | 2202.1 | 2041.9–2370.1 | <0.001 | 1227.6 | 1125.8–1333.8 | <0.001 |
| Cancer | 1292.3 | 1261.0–1324.1 | <0.001 | 1076.3 | 1051.7–1102.3 | <0.001 | 2621.0 | 2557.1–2686.1 | <0.001 | 2184 | 2133.0–2235.8 | <0.001 |
| Joint disease | 213.7 | 188.0–240.1 | <0.001 | 104.1 | 84.7–124.4 | <0.001 | 470.5 | 416.2–526.5 | <0.001 | 234.4 | 193.6–276.4 | <0.001 |
| Weight loss | 262.6 | 231.0–295.3 | <0.001 | 6.9 | -13.0–27.5 | 0.500 | 591.0 | 523.7–661.0 | <0.001 | 36.0 | -5.4–78.9 | 0.089 |
| Fluid and electrolyte disorders | 689.2 | 583.7–803.8 | <0.001 | 135.2 | 80.8–193.5 | <0.001 | 1590.4 | 1358.8–1841.9 | <0.001 | 352.9 | 235.3–479.4 | <0.001 |
| Anemia | 936.5 | 853.0–1024.8 | <0.001 | 358.5 | 312.4–407.6 | <0.001 | 2001.3 | 1824.9–2187.7 | <0.001 | 754.5 | 657.9–855.8 | <0.001 |
| Clotting disorder | 1653.1 | 1370.2–1975.4 | <0.001 | 1581.8 | 1338.2–1855 | <0.001 | 4045.7 | 3391.5–4789.7 | <0.001 | 4009.4 | 3432.1–4656.1 | <0.001 |
| Depression/Addiction | 298.1 | 181.0–431.7 | <0.001 | 268 | 168.6–379.8 | <0.001 | 657.1 | 408.3–940.5 | <0.001 | 580.5 | 372.4–813.4 | <0.001 |

^a^The estimates were obtained from the multivariable gamma regression

^b^Reference groups as no preoperative complications.

All estimates were calculated using an unmatched sample

**Supplemental Table 3: Impact of postoperative sepsis complication, social-demographic characteristics, and comorbidities on readmission and reexamination**

| **Factors** | **Readmission** | | **Reexamination** | |
| --- | --- | --- | --- | --- |
|  | **OR** | **95%CI** | **OR** | **95%CI** |
| **Postoperative sepsis** | 6.40^***^ | 6.06–6.76 | 1.67^***^ | 1.58–1.76 |
| **Age** | 1.01^***^ | 1.01–1.01 | 1.00 | 1.00–1.00 |
| **Gender** *(Ref: Male)* |  |  |  |  |
| Female | 0.85^***^ | 0.84–0.86 | 1.06^***^ | 1.05–1.07 |
| **Region** *(Ref: Northern Midlands and Mountainous)* |  |  |  |  |
| Red River Delta | 0.86^***^ | 0.84–0.88 | 1.31^***^ | 1.29–1.33 |
| North Central and South Central Coast | 0.82^***^ | 0.80–0.84 | 1.86^***^ | 1.83–1.89 |
| Central Highland | 0.83^***^ | 0.81–0.86 | 2.74^***^ | 2.68–2.79 |
| Southeast | 0.60^***^ | 0.58–0.61 | 5.13^***^ | 5.05–5.21 |
| Mekong River Delta | 0.63^***^ | 0.62–0.65 | 4.93^***^ | 4.84–5.01 |
| **Emergency hospitalization** | 1.12^***^ | 1.10–1.14 | 1.00 | 0.99–1.01 |
| **Preoperative concomitant disease** |  |  |  |  |
| Heart failure | 1.02 | 0.95–1.11 | 0.95 | 0.90–1.00 |
| Valvular heart disease | 1.17^**^ | 1.06–1.30 | 1.91^***^ | 1.78–2.05 |
| Peripheral vascular disease | 1.23^**^ | 1.08–1.39 | 1.08 | 0.98–1.18 |
| Arrhythmia | 1.09^*^ | 1.01–1.18 | 0.91^***^ | 0.86–0.96 |
| Hypertension | 1.13^***^ | 1.10–1.16 | 1.75^***^ | 1.72–1.78 |
| Cerebrovascular disease | 1.11^***^ | 1.07–1.16 | 0.95^***^ | 0.92–0.97 |
| Paralysis | 2.17^***^ | 1.84–2.55 | 0.83^**^ | 0.72–0.95 |
| Chronic lung disease | 1.22^***^ | 1.16–1.28 | 1.20^***^ | 1.16–1.24 |
| Diabetes | 1.22^***^ | 1.17–1.26 | 2.08^***^ | 2.03–2.13 |
| Complicated diabetes | 1.06 | 0.84–1.33 | 1.53^***^ | 1.28–1.83 |
| Gastritis | 1.25^***^ | 1.22–1.28 | 1.38^***^ | 1.36–1.40 |
| Hypothyroidism | 0.98 | 0.81–1.18 | 1.63^***^ | 1.45–1.83 |
| Chronic renal failure | 3.22^***^ | 3.03–3.42 | 1.24^***^ | 1.17–1.31 |
| Liver disease | 1.08^***^ | 1.04–1.13 | 1.13^***^ | 1.09–1.16 |
| Metastatic cancer | 2.35^***^ | 2.19–2.52 | 1.19^***^ | 1.12–1.26 |
| Cancer | 5.50^***^ | 5.38–5.62 | 1.15^***^ | 1.12–1.17 |
| Joint disease | 0.95 | 0.89–1.01 | 1.43^***^ | 1.38–1.49 |
| Weight loss | 1.07 | 1.00–1.14 | 1.17^***^ | 1.12–1.23 |
| Fluid and Electrolyte Disorders | 1.09 | 0.94–1.27 | 0.63^***^ | 0.57–0.71 |
| Anemia | 1.41^***^ | 1.28–1.54 | 1.01 | 0.94–1.08 |
| Clotting disorder | 1.52^***^ | 1.20–1.92 | 1.11 | 0.93–1.33 |
| Depression/Addiction | 1.30 | 0.99–1.71 | 1.50^***^ | 1.25–1.78 |

95% CI: 95% Confidence interval; All estimates were calculated using unmatched sample

*^***^ p < 0.001; ^**^ p < 0.01; ^*^ p < 0.05*
